# What Psychiatric Interventions Are Used for Anxiety Disorders in Infertile Couples? A Systematic Review Study

**Published:** 2019-04

**Authors:** Seyedeh Zahra Masoumi, Parisa Parsa, Fatemeh Kalhori, Hossein Mohagheghi, Younes Mohammadi

**Affiliations:** 1 Mother and Child Care Research Center, Hamadan University of Medical Sciences, Hamadan, Iran.; 2 Chronic Diseases (Home Care) Research Center, Hamadan University of Medical Sciences, Hamadan, Iran.; 3 Consultation in Midwifery, Department of Midwifery, Hamadan University of Medical Sciences, Hamadan, Iran.; 4 Faculty of Economic and Social Science, Bu-Ali Sina University, Hamadan, Iran.; 5 Modeling of Noncommunicable Diseases Research Center, Department of Epidemiology, School of Public Health, Hamadan University of Medical Sciences, Hamadan, Iran.

**Keywords:** *Anxiety*, *Assisted Reproductive Technology*, *Infertility*, *Psychosocial Interventions*

## Abstract

**Objective:** Infertility causes psychological and social problems in many infertile women, men, and couples, and the most common of which is anxiety. Also, assisted reproductive treatments (ART) increase anxiety. Numerous medical and community associations have strongly recommended psychosocial interventions and counselling to help infertile couples.

**Method** **:** A review was done on studies published from 1982 to 2018 that were indexed in Medline, ISI Web of Knowledge, and Scopus. The references of each article were used for more resources and references.

**Results: **Most psychosocial interventions were effective on infertile women, men, and couples. These articles showed a significant decrease in anxiety with CBT interventions, body-mind interventions, and other interventions.

**Conclusion: **All interventions based on CBT, most interventions based on body-mind, and some other interventions are effective in treating anxiety in infertile women and men.

Infertility means not having children after one year of regular sexual life without using contraception techniques (1). According to the World Health Organization (WHO) 8%–10% of couples experience difficulty in conceiving. Infertility causes psychological trauma for most couples, often experienced as the most stressful event in their lives (2). While infertility is not a disease, it and its treatment can affect all aspects of people’s lives, which can cause various psychological-emotional disorders or consequences, including turmoil, frustration, anxiety, anxiety, hopelessness, guilt, and feeling of worthlessness in life (3). Fecundity has become a growing problem for many couples trying to conceive a child, and although not all couples choose to seek medical assistance, more than 10% of the child-bearing population have used assisted reproductive technology (ART) to conceive (4, 5).

Men and women undergoing ART experience high stresses related to both the experience of infertility and participation in associated medical treatments (6). The provision of psychosocial interventions for infertile couples has been recommended since the advocacy work of Barbara Eck directed research attention to emotional distress as a consequence of infertility rather than, as had been the emphasis until then, a cause of infertility (7). According to Boivin, positive results from psychological interventions can occur in feelings of anxiety, tension, and worry more than in depressive symptoms (8). Many studies have been conducted on the necessity and importance of psychological interventions and counseling for infertile couples (9, 10, 11).

An updated review is needed to determine what psychosocial interventions may affect infertility-related anxiety. In addition, it is important to review the outcomes of studies that conducted psychosocial intervention to treat anxiety in infertile men and women. 

## Materials and Methods

This systematic review was performed in 2018 to review all studies published from 1982 to 2018. Because of different tools and types of interventions, contradictory results, and high heterogeneity of the studies, no meta-analysis was done. These articles were indexed in Medline, ISI Web of Knowledge, and Scopus. Also, abstract books on this subject, regardless of type of study, its publication status, language, sex of individuals, or treatment protocols, were included in this review. We also corresponded with the authors of cited references for more resources and references. The research strategy involved general and specific terms related to infertile women, including "psychology", "psycological", "intervention", "method", "methodological", "procedure", "hypervigilance", "nervousness", "social anxiety”, ”anxiety” "infertility" and "sterility". The main inclusion criteria were as follow: assessment of psychosocial interventions multidimensionally using general or specific interventions on anxiety; use of standard interventions assessing psychosocial interventions on anxiety in infertile men and women. All procedures were performed by two independent researchers and any disagreement between them was evaluated by interviewing them and then by feed-back from a third person. The abstracts and full-texts of the papers were primarily reviewed for rejection or acceptance. Then, the full-texts of the accepted papers were critically evaluated and the needed data were extracted. Because the tools and types of interventions were different, contradictory results were observed, and heterogeneity of the studies was high, no meta-analysis has been used. The authors' names and those of the journals and the results were concealed from the researchers. To identify interventions on anxiety in infertile men and women, Medical Outcomes Trust (12) was used, which includes a set of attributes and criteria: (1) conceptual and measurement models; (2) reliability; (3) validity; (4) responsiveness; (5) interpretability; (6) respondent and administrative burden; (7) alternative forms; and (8) cultural and language adaptations (translations). The interventions have been presented in Table 1.

## Results

**Figure 1:**
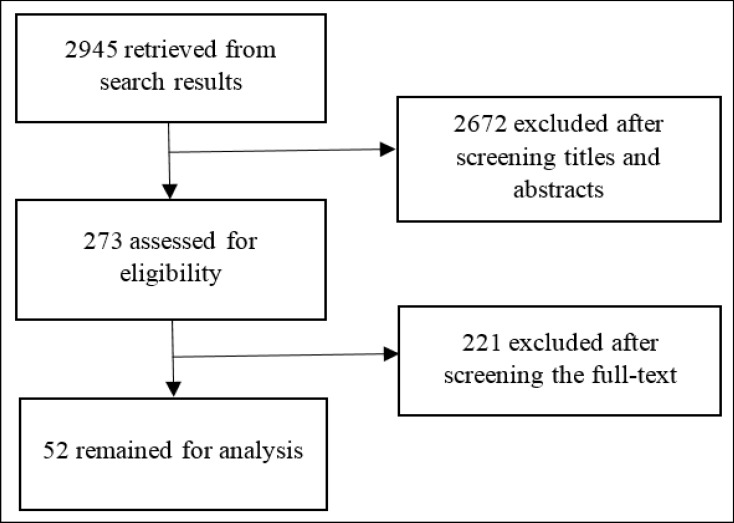
Flow Diagram of the Progress through the Phases of Systematic Review

Among 2945 studies obtained from physical and electronic resources, some studies were repetitive and some found to be irrelavant after reviewing their abstracts or full-texts, and thus were excluded. In total, 52 studies were reviewed and analyzed (Figure 1).

After initial assessment and comparison of the results, the papers were categorized based on the following criteria: (a) design of interventions; (b) quantitative assessment of psychological interventions in different cultures; (c) assessment of anxiety in patients under treatment and evaluation of the effects of therapeutic interventions on improving quality of life; and (d) assessment of the impact of psychological interventions on anxiety in infertile couples. Psychological interventions that are frequently used for anxiety in infertile men and women are as follow:

(A) CBT interventions; (B) Body-Mind Interventions; and (C) Other Interventions

***CBT interventions***

Cognitive-behavioral therapy (CBT) has been used successfully to resolve or reduce anxiety in infertile women (13). Several studies have used cognitive behavior therapy in infertility counseling. 

Domar used cognitive-behavioral therapy for infertile women. Participants in the CB group met for 2 hours on a weekly basis for 10 weeks on a weekday evening and they were introduced to a wide variety of techniques, including relaxation-response training, cognitive restructuring, emotional expression, and nutrition, and exercise information relevant to infertility (14). 

In the study of Heydari conducted on infertile women undergoing IUI, cognitive behavioral therapy was applied in the experimental group individually and face-to-face for 10 minutes and included information about the anatomy of the reproductive system, sperm and ovum, physiology of the fetus, how to perform IUI steps, anxiety symptoms and its effect on fertility, physiological changes during anxiety, and relaxation. Also, focus technique training and breathing were taught for 10 minutes. Then, each participant practiced the technique and persisted to acquire the skills. Participants have to do the breathing practice 3 times a day for 10 minutes at home and note them on the sheet (15).

In another research that was performed on infertile women, the therapist considered social, sexual, and relationship concerns of the infertile women for restructuring and eliminating in CBT sessions. The first 3 sessions provided patients with a general orientation to cognitive therapy and the causes of infertility. A gynecologist participated in these sessions for 30 minutes and explained the cause of infertility for each patient. Fourth and fifth sessions included the identification and challenges of core dysfunctional or irrational beliefs that underlie automatic negative thoughts about infertility. Finally, in sessions 7–10 participants were taught various techniques (eg, countering, self-reward) to change their dysfunctional beliefs about infertility. In addition to the above program, in sessions 5–10 participants were taught progressive muscle relaxation in a group setting (16).

In a study performed on infertile couples by Noorbala, cognitive-behavioral therapy included recognizing negative thinking to help the participants distinguish phobia from reality and thereby change their cognitive structure. The behavioral techniques used included physical activity (daily walking), muscle relaxation exercises, imagination exercises, expressing feelings, keeping a balanced diet, and engaging in leisure activities (13).

A study was conducted by Hamzehpour on infertile women, in which he focused on cognitive behavioral education, including emphasis on attitude, identification and rehabilitation of cognitive distortions, correct thinking techniques, and recognizing judgments and negative thoughts that have an important effect on mental health of infertile women and help reduce anxiety levels and other psychological problems in these women (17). CBT builds a set of skills that enables an individual to be aware of thoughts and emotions, to identify how situations, thoughts, and behaviors influence emotions, and to improve feelings by changing dysfunctional thoughts and behaviors (18, 19).

***Body-Mind Interventions***

Chan conducted a study on anxiety of infertile women. In their study, Eastern Body-Mind-Spirit was used. 

Group intervention took place at the Centre on Behavioral Health, University of Hong Kong and consisted of 4 weekly sessions that were held on Saturday or Sunday evenings.

Under the EBMS framework, physical, psychosocial, and spiritual well-being are believed to be interconnected and all play a major role in the face of stressful life events. (20). 

In a study, Anna Galhardo used Mindfulness-Based Program (MBPI) method on infertile women with psychological problems. The MBPI is based on Mindfulness-Based Program for stress reduction, the Mind/Body Program for Infertility, and basic principles of acceptance and commitment therapy (ACT). It is a structured group intervention targeting infertile women that aims to cultivate mindfulness and acceptance, helping them to move towards a chosen and valued life direction. One of the main goals of MBPI is to promote psychological flexibility/acceptance. After the first half hour, a formal mindfulness practice is held, followed by sharing how participants felt, what they have noticed, and how the experience was for them. The set of formal practices selected for the MBPI is commonly used in mindfulness programs. Informal mindfulness practice is also presented as early as the second session through mindful eating. Metaphors and experiential exercises are included in most of the sessions, which include an experiential exercise of listening to others, introduction of values clarification through imagery exercise, and a psychoeducational section on healthy lifestyle (exercise, nutrition, caffeine, alcohol, nicotine, herbal remedies, etc).

A trained yoga teacher conducts this practice following a sequence of yoga postures recommended by Kabat-Zinn in the Mindfulness-Based Stress Reduction Program. Before lunch, participants watch the video ‘The Joy of Stress by Loretta LaRoche’. In the afternoon, creativity is encouraged through the ‘‘map of life” exercise. Although this exercise resembles that of the Mind/Body Program for Infertility, its aim is to highlight the importance of values as the chosen life directions and to commit to building patterns of effective action towards a meaningful life. After this, each couple is invited to accomplish a communication exercise intended to provide an opportunity for each partner to share her/his feelings about the relationship (8).

Hoveyda conducted mind-body-based interventions on infertile women with mindfulness-based stress reduction (MBSR) and conscious yoga. MBSR was presented by Kabat-Zinn in Medical Centre of Massachusetts University in 1979. It is an 8-week program and each session lasts 120 to 180 minutes. Mindfulness skills are taught to coping with life stresses and raise awareness about the present moment and include thought-related meditation, relaxation, and Hatha yoga. The sessions introduced the following strategies: automatic guidance system, learning how to use the present moment, awareness of bodily sensation, thoughts and emotions in reducing stress, practicing eating raisins, giving feedback and discussion about the practice, 3-minute breathing, practicing breathing mindfulness meditation, yoga stretching exercise, having conscious sitting with awareness of breathing , 5-minute practicing of “seeing or hearing”, re-practicing conscious session with awareness of breathing and body, re-practicing conscious session , explaining stress and identifying participants’ reactions to stress, examining awareness of pleasant and unpleasant events on feelings, thoughts, and bodily sensations, mindfulness of sounds and thoughts, practicing mountain meditation, sleep hygiene, making a list of enjoyable activities, examining and discussing programs, practicing stone, and beads and marbles meditation (21).

***Other interventions***

The third category of articles are related to psychosocial interventions that have been called “other” interventions in Table 1. They include emotion-focused and problem-focused coping, counselling (psychological support), psychotherapy, supportive psychotherapy, experiential psychosocial therapy, interacting cognitive subsystem, supportive psychotherapy, online psychoeducational support, supporting stress management, cognitive coping, and relaxation intervention, group positive psychotherapy, and group reality therapy.

Debra used emotion-focused and problem-focused group therapies for women with fertility

Problems. Problem-focused coping demands include the necessity for seeking information regarding diagnostic and treatment options, actively pursuing treatments designed to improve fertility, and assertively communicating with partners and medical personnel to increase the probability of successful treatment. Emotion-focused treatment had 3 primary goals: (1) to encourage emotional expression surrounding fertility concerns, (2) to promote pleasurable activities and relaxation to counteract the experience of negative infertility-related emotions, and (3) to reduce negative affect associated with dysfunctional beliefs surrounding infertility. Primary aims of problem-focused techniques were to increase perceived control and mastery over infertility through interacting assertively about infertility issues with medical personnel and others, increasing sources of infertility-relevant information, and promoting problem-solving strategies on infertility concerns. Potentially useful emotion-focused strategies include expression and processing of feelings surrounding the experience, implementation of strategies designed to ameliorate negative emotions, and enhancement of communication skills to facilitate emotional expression and emotional support-seeking (22).

Another type of intervention was support group for infertile women in Domar study. Support groups are the most common psychological intervention offered to infertile women (14).

The aim of the Emery study was to evaluate the RMU's model of routine pre-IVF counselling, which focuses on the narrative capacities of couples. However, this model of counselling focusing on narrative is clearly focused on psychological support. The hypotheses were that routine pre-IVF is acceptable to most couples, and that it can contribute to decreasing anxiety and depressive symptoms during and after the first cycle of IVF, and that couples feel they are helped through this form of psychological assistance (23).

In Yektatalab study, infertile women were trained with group psychotherapy in the experimental group. The intervention took 12 two-hour sessions, with 5-day intervals. Subjects of psychotherapy included discussion and education on the causes and diagnostic and treatment methods used in infertility, psychological aspects of infertility, the impact of anxiety on the reproductive system, and the process of crisis. (24).

In Klerk study, the intervention consisted of 3 sessions, with a social worker trained in experiential psychosocial therapy: one before, one during, and one after the first IVF cycle. The objective of Klerk study was to evaluate a psychosocial counselling intervention for first time IVF couples. The main goal of experiential psychosocial therapy is teaching clients new skills by forming not only a professional but also a personal relationship with them. Instead of being an objective observer, the counsellor expresses his/her own feelings and ideas about the client to create new interpersonal experiences for that client. It is assumed that through these personal experiences with the therapist clients learn how to cope with interpersonal problems (25).

Two articles of Nilforooshan were about cognitive-behavioral counseling based on interacting cognitive subsystems (ICS). ICS proposes that implicational meaning plays a crucial role in the production of emotion. ICS approach emphasizes the close relationship between implicational level and states of bodily arousal. Issues about infertility, anxiety, feelings and thoughts, schemas and how they are designated and recognizing them, and creating substituted schemes with control schemas were discussed. Relaxation and concentration on breathing and mindfulness techniques were practiced. At the first session of ICS, the participants were informed about goals, definition of infertility, anxiety generation and its symptoms, and the relationship between anxiety and infertility. In addition, relaxation practice along with mental imagery was done. The second session was allocated to the identification of thoughts and feelings, and the focus was on working on feelings directly and their acceptance without judgment, and concentration on breathing.

The third session focused on thoughts. The fourth session started with mindfulness, followed by identifying schematic models and developing new schematic models to replace the former ones. Concentration on breathing was practiced. The fifth session was allocated to developing the control schema, and the main focus was on how they can take care of themselves. At the sixth session, they were taught to cope with their mood status in future using their instructions (26, 27).

In the study of Noorbala, supportive psychotherapy intervention has been evaluated (A) the suitability of the psychological treatment, the cause of infertility, and the most suitable infertility treatment for each couple; (B) the depressed participants' psychological and emotional responses to family, friends, and others; and (C) the depressed participants' selfesteem in their relation to partner, friends, colleagues, and others (13).

Cousineau used online psychoeducational support for infertile women to form of Solomon-four group design. This assessment serves as the tailoring mechanism of the online program, resulting in targeted feedback based on a high, medium, or low confidence level in the areas of taking care of oneself, managing feelings, and relationship with partner; managing treatment, and relationship with health care provider (28).

In Mori study, supporting stress management was performed for women undergoing the early stages of fertility treatment. Stress management education is stress-related health education with the intention of allowing healthy people to avoid becoming unhealthy and to improve their health. It helps people to recognize stress and to learn how to control stressors and stress responses. Stress management education is stress-related health education with the intention of allowing healthy people to avoid becoming unhealthy and to improve their health. It helps people to recognize stress and to learn how to control stressors and stress responses. The participants used notebooks as a stress diary, a relaxation diary, a social support network, and a stress calendar (29). 

In the Hakim study, psychological readiness has been used for women who use assisted reproductive techniques. The broad purpose of the session was to discuss the implications of pursuing IUI treatment and specific topics addressed included the role and availability of counselling services, the psychosocial and infertility history, potential concerns about IUI treatment, the experience of infertility-related stress on both individuals and the relationship, the utilization of coping strategies, availability and use of social support, and any other stressors of concern. Counsellors addressed all topics to some extent but tailored the session to each couples’ expressed concerns. The counselling session typically lasted 60–90 minutes (30).

Nekavand study was about the effect of relaxation on infertile women undergoing IVF treatment. Technique of progressive muscle relaxation is used to reduce muscle tension or muscle contraction. 

Progressive muscle relaxation technique is a practice with a series of systematic steps that helps the patients to contract a group of muscles regularly and then relax them. This method neutralize muscle tension. Progressive muscle relaxation increases activity of the parasympathetic cycle and neutralizes muscle tension. This method is done through traction contrasting modes and relaxing muscles (31). 

In Domar research, in vitro fertilization (IVF) patients in group of cognitive coping and relaxation intervention (CCRI) were compared for emotions, quality of life, discontinuation, and pregnancy rates with routine care (RC). The CCRI comprised a cognitive and a relaxation component adapted for the study from 2 existing support interventions to help women cope with the stimulation and waiting phases of ART. The relaxation component of the CCRI consisted of relaxation techniques taken from the Mind/Body Program (MBP) for infertility. The cognitive component of the CCRI was positive reappraisal coping intervention. The PRCI intervention consists of an explanatory leaflet and a set of 10 statements designed to facilitate the use of positive reappraisal coping, a form of coping that helps people think more about the positive aspects of a difficult situation and dwell less on its negative aspects. (32). 

Seyedi Asl worked on positive psychotherapy in infertile woman. It is one of the new approaches in psychology, which is innovated for treating psychological disorders and enhancing positive emotions. The aim of that study was to investigate the effectiveness of group positive psychotherapy on increasing life satisfaction and quality of life in infertile women. The treatment of the control group was delayed by 6 weeks, but the intervention group participated in a 6-week group positive psychotherapy. Each session lasted 90 minutes and was held in a group therapy format. In each meeting, tasks were completed by the participants for the next meeting. The meetings included the following 6 positive exercises: (i) using strengths; (ii) gratitude visit; (iii) active-constructive response; (iv) counting blessings; (v) savoring; and (vi) biography. The subjects of sessions were as follow: opening and positive introductions; using strengths; gratitude letter; active-constructive responses; blessings; write a short essay; biography and savoring (33).

Soltanzadeh Mezreji study examined the efficacy of group reality therapy on infertile women undergoing treatment with assisted reproductive techniques (IUI). Group reality therapy in the experimental group was administered over 10-90-minute sessions. Tools of reality therapy include intense emotional relationship of the individual, facing the facts, abandoning irresponsible behaviors, and training to adapt a better behavior. The sessions covered the following issues: implementation of the pretest and diagnostic interview; familiarity of members with the concept of reality therapy and emotional interaction with group members; members’ familiarization with their own identity; types of identity and characteristics of a successful and unsuccessful identity; how to take responsibility for one’s behavior; and importance and necessity of responsibility in life. Moreover, the sessions focused on the following topics: introduction to anxiety from the perspective of reality therapy and anxiety management skills training; familiarization with basic and effective needs in real life; familiarization with how to plan problem-solving; planning for life and for the treatment assisted reproductive method IUI; familiarization with the commitment to implement medical programs; and familiarization with denying excuses on implementing selected projects and programs (34).

The objective of Moeenizadeh study was to examine the effectiveness of well-being therapy (WBT) on anxiety of infertile women. WBT is based on an educational model; it is structured, directive, and problem-oriented and presents problems and situations. WBT includes technique of self-observation along with the use of a structured diary and interaction between patients and therapists. The therapy sessions are divided into 3 phases: initial, intermediate and final. These sessions identify incidences of well-being and apply the rules into situational context regardless of its short period (17). The patients were also asked to give a daily report in which the circumstances of well-being incidences ranged from 0-100, with zero indicating lack of well-being and 100 full well-being (35).

**Table 1:** Psychosocial Intervention and Sample Size of the Reviewed Studies

**Author**	**Country**	**Participants (N)** **I: intervention** **C: control** **W: women** **M: men**	**Study design**	Intervention type*	**Intervention** **category**	**Format**	**Number of sessions**	**Duration** **(weeks)**	**Follow up**
Debra A et al (1997)	USA	N=29W I=20W C=9W	(Pretest-posttest)	Emotion- focused and Problem-focused coping	Other	Group	6	6	1 & 18 Months
Alice D. Domar et al (2000)	USA	N=184W I_1_=56W I_2 _=65W C=63W	Randomized clinical trial	Cognitive Behavioral Therapy & support group	CBT & Other	Group	10	10	6 and 12 months
Heydari P et al (2002)	Iran	N=110W I=55W C=55W	Randomized clinical trial	Cognitive Behavioral Therapy	CBT	Group	12-13		
M.Emery et al (2003)	Switzerland	N=282W&M (141couple) Group A=100 Group B=94 Group C=30 Group D=58	Prospective, randomized, controlled study	Counselling (Psychological support)	Other	Group			
Yektatalab Sh et al (2004)	Iran	N=60W I=30W C=30W	Semi experimental	Psychotherapy	Other	Group	12	10	
C.de Klerk et al (2005)	The Netherlands	N=84W&M(couple) I=43W&M C=41W&M	Randomized controlled trial	Experiential Psychosocial Therapy	Other	Group	3		
Celia H. Y et al (2006)	China	N=227W I=69W C=115W	Randomized controlled study	(EEastern Body -MMind- Spirit)	E EBMS	Group	4	4	
Nilforooshan P et al (2006)a	Iran	N=30W&M(couple) I=15W&M C=15W&M	Quasi-experimental	Interacting Cognitive Subsystem	ICS (Other)	Group	6	6	2 Months
Nilforooshan P et al (2006)b	Iran	N=30W&M(couple) I=15W&M C=15W&M	Quasi- experimental	Interacting Cognitive Subsystem	ICS (Other)	Group	6	6	
Faramarzi M et al (2007)	Iran	N=59W I=29W C=30W	Randomized controlled clinical trial	Cognitive Behavioral Therapy	CBT	Group	10	10	
Ahmad A. Noorbala et al (2008)	Iran	N=140W&M(couple) I_1_ =70W&M I_2 _=70W&M	Cross-sectional study	Cognitive-Behavioral Therapy & Supportive psychotherapy	CBT & Other	Group			6Months
Tara M. Cousineau et al (2008)	USA	N=190W I_1_ =50W I_2_ =47W C_1_ =49W C_2_ =44W	Randomized controlled trial	Online psychoeducational support	Other	Group	2		4weeks
Hamzehpourhaghighi T et al (2009)	Iran	N=30W I=15W C=15W	Experimental (Pretest-posttest)	Cognitive Behavioral Therapy	CBT	Group	8	8	
Akiko Mori(2009)	Japan	N=140W I=96W C=44W	A cluster randomized Controlled trial50	Supporting stress management	Other	Individual			1& 2& 3 Months
Lila Z. Hakim et al (2012)	Cananda	N=83W&M(couple)	Semi-structured	Preparatory psychosocial counselling	Other	Individual/ couple	1		
Ana Galhardo et al (2013)	Portugal	N=55W I=18W C=37W	Controlled clinical trial	Mindfulness-Based Program for Infertility	MBPI	Group	10	10	
Hoveyda Sh et al (2014)	Iran	N=24W I=12W C=12W	Randomized controlled trial	Mindfulness- Based Stress Reduction program and group conscious yoga	MBSR	Group	8	8	2 Months
Nekavand et al (2014)	Iran	N=100W I=50W C=50W	Experimental (Pretest-posttest)	Relaxation	Other	Group	3		
Hamzehpour T et al (2014)	Iran	N=30W I=15W C=15W	Experimental (Pretest-posttest)	Cognitive Behavioral Therapy	CBT	Group	8	8	
Talaei A et al (2014)	Iran	N=20W I=10W C=10W	Interventional (Pretest-posttest)	Cognitive Behavioral Therapy	CBT	Group	10	10	
Alice D. Domar et al (2015)	UK	N=166W I =89W C =77W	Randomized, controlled, prospective study	Cognitive Coping and Relaxation Intervention	CCRI (Other)	Group			12 months
Seyed Teymur Seyedi Asl et al (2016)	Iran	N=36W I =18W C =18W	Randomized trial study(Pretest-posttest)	Group positive psychotherapy	Other	Group	6	6	
SoltanzadehMezreji H et al (2016)	Iran	N=40W I =20W C =20W	semi-experimental (pretest-posttest)	Group reality therapy	Other	Group	over 10		
Moeenizadeh M et al (2016)	Iran	N=22W I =11W C =11W	preliminary trial	Well-Being Therapy	WBT (Other)	Group	8 - 10	8	

## Discussion

This study reviewed the effectiveness of psychosocial interventions on anxiety and depressive symptoms of couples. In all studies of category A (CBT method), the samples were women, with the exception of a study, which was conducted on couples. Also, all studies showed reduction in anxiety in this category. There was a study in category B with different results. 

Some studies used mindfulness (mind-body method) as an intervention. In category C, counselling (psychological support), experiential psychosocial therapy, group positive psychotherapy, and supporting stress management methods did not have any effects on reducing anxiety, but they were effective in other studies (23, 25, 29, 33). 

Most psychosocial interventions used cognitive-behavioral therapy mothod and the least interventions were in category. In the present study, we found that the majority of psychosocial interventions are effective in reducing anxiety in infertile women, men, or couples. These studies used several methods: cognitive-behavioral therapy (13-19); Eastern body-mind spirit (24); interacting cognitive subsystem (26, 27); online psychoeducational support (28); relaxation (31); group reality therapy (34); support group (14); psychotherapy (24); preparatory psychosocial counselling (30); mindfulness-based stress reduction program (8); mindfulness-based program for infertility, group conscious yoga (21); well-being therapy (35); cognitive coping and relaxation intervention (36); emotion-focused and problem-focused coping (22), group positive psychotherapy (33); and supportive psychotherapy (36, 37). Finally, many of the reviewed studies showed that psychosocial interventions have a positive effect on anxiety in infertile patients. 

## Limitation

In this study, only articles in English were used. Also, unpublished and gray literatures were not used in the study.

## Conclusion

Based on the results of this study, psychological interventions for anxiety are necessary for infertile couples undergoing fertility treatment. There are several interventions for treating anxiety in this group, such as CBT interventions and body-mind interventions.
